# Interpretable machine learning analysis of environmental characteristics on bacillary dysentery in Sichuan Province

**DOI:** 10.3389/fpubh.2025.1598247

**Published:** 2025-07-16

**Authors:** Yao Zhang, Qiao-Lin Wang, Wei Peng, Meng-Yuan Zhang, Yao Qin, Lun Zhang, Rong-Jie Wei, Dian-Ju Kang

**Affiliations:** ^1^Department of Emergency Management, Sichuan Center for Diseases Control and Prevention, Chengdu, China; ^2^West China School of Public Health/West China Fourth Hospital, Sichuan University, Chengdu, China; ^3^Department of Health Education Institute, Sichuan Center for Diseases Control and Prevention, Chengdu, China

**Keywords:** bacterial dysentery, climate zones, environmental characteristics, XGBoost, SHAP

## Abstract

**Background:**

Bacterial dysentery (BD) is a leading cause of diarrhea-related mortality globally, with its incidence heavily influenced by environmental factors. However, a climate zone-specific predictive model for BD was currently lacking in Sichuan Province.

**Objective:**

This study aims to employ interpretable machine learning to explore the influence of environmental factors on BD incidence across different climate zones and to elucidate their interaction mechanisms.

**Methods:**

Monthly data on meteorological and ecological factors, along with BD case reports, were collected from 183 counties in Sichuan Province (2005–2023). The eXtreme Gradient Boosting (XGBoost) algorithm was employed to assess the influence of key environmental features, including precipitation, temperature, PM10, potential evaporation, vegetation cover, and NDVI, on BD incidence. To enhance interpretability, the model’s outputs were visualized and explained using SHapley Additive Explanations (SHAP).

**Results:**

A machine learning model was developed to assess the impact of environmental factors on BD incidence across different climate zones. The findings revealed significant spatial heterogeneity in key drivers of BD. In the Central Subtropical Humid Climate Zone, BD incidence was predominantly influenced by average temperature, PM10, and minimum temperature. In the Subtropical Semi-Humid Climate Zone, potential evaporation, PM10, and precipitation emerged as the primary determinants. In the Plateau Cold Climate Zone, PM10, minimum temperature, and precipitation were the most significant factors. Notably, PM10 consistently showed a positive correlation with BD across all climate zones. Furthermore, average temperature showed a positive association with BD in the Central Subtropical Humid Climate Zone, while potential evaporation and minimum temperature demonstrated similar positive relationships in the Subtropical Semi-Humid and Plateau Cold Climate Zones, respectively. Additionally, precipitation displayed a U-shaped relationship with BD risk in both the Subtropical Semi-Humid and Plateau Cold Climate Zones.

**Conclusion:**

This study developed a climate zone-specific predictive model for BD, systematically evaluating the interactions between environmental factors and BD dynamics. The findings provide a scientific basis for refining targeted public health intervention strategies.

## 1 Introduction

Bacterial dysentery (BD), caused by Shigella, is an intestinal infectious disease transmitted through contaminated food, water, and person-to-person contact (1). It poses a significant public health challenge globally, particularly in developing countries (2). Although the incidence of BD has been effectively reduced in many regions worldwide over the past few decades through improved sanitation and public health interventions, it remains one of the leading causes of diarrheal mortality globally (3). According to statistics, BD caused 210,000 deaths in 2016, with over 90% of cases occurring in developing countries, particularly among children under 5 years old and adults over 70 years old (4, 5).

BD exhibits distinct seasonal and geographical patterns. The peak periods of BD vary significantly across regions. For instance, in Bangladesh, the peak typically occurs between September and November (6); in Vietnam, it is between May and October (7); and in Sweden, between July and October (8). Studies have shown that temperature and precipitation directly influence pathogen survival and transmission (9, 10), highlighting the role of meteorological factors. In China, due to regional differences in meteorological conditions, the transmission patterns and peak periods of BD also vary (11, 12). Northern regions typically experience peaks in early summer, while southern regions experience peaks in summer and autumn (13, 14).

Recent extreme weather events, such as floods and El Niño, have exacerbated BD outbreak risks (15, 16). These events often lead to abnormal temperature increases, which are associated with BD epidemics (17, 18). Notably, in addition to meteorological conditions, ecological factors also play a significant role in BD incidence. For example, increased forest cover can help prevent BD by improving water quality (19).

Although many studies have examined the relationship between meteorological factors and BD, most relied on single-factor analyses and short-term, large-scale data (20–23), limiting understanding of local transmission patterns and characteristics. The impact of local meteorological and ecological factors on disease dynamics may differ significantly from findings based on large-scale studies (24, 25). Furthermore, short-term data cannot reflect long-term trends. Therefore, developing models that integrate long-term, fine-scale meteorological and ecological data is crucial for predicting and analyzing BD activity.

Traditional statistical methods are limited in capturing the complex nonlinear relationships between meteorological, ecological, and disease incidence data (26). Therefore, adopting more advanced machine learning methods can more effectively capture these complex relationships, providing more accurate predictions and assessments for BD prevention and control. As an efficient machine learning method, eXtreme Gradient Boosting (XGBoost) has demonstrated significant potential in various fields in recent years, particularly in handling large-scale data and modeling nonlinear relationships among multidimensional variables, offering greater flexibility and accuracy compared to traditional regression models (9, 27). It can efficiently process high-dimensional, nonlinear, and heterogeneous complex data and, through the integration of multiple decision trees, exhibits strong predictive capabilities, accurately capturing the relationships between diseases and multiple influencing factors (28, 29). This provides a scientific basis for the formulation of health intervention strategies (30, 31). Therefore, this study employs the XGBoost model to assess the impact of meteorological and ecological factors on BD activity across different climate zones at the county and monthly scales, providing data for targeted public health interventions.

## 2 Materials and methods

### 2.1 Study area

**Figure 1:**
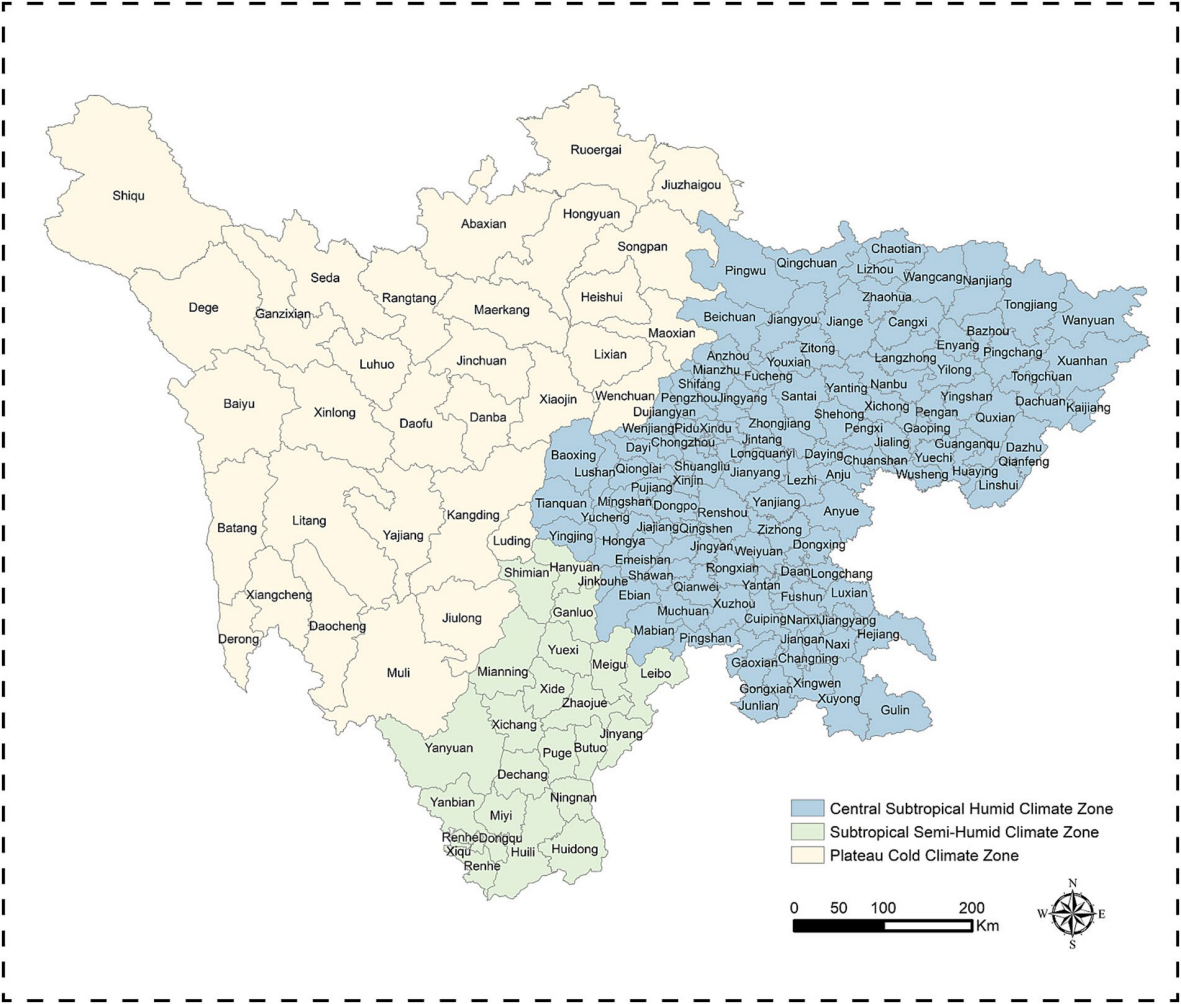
Spatial distribution of climate zones and 183 county-level administrative divisions.

Sichuan Province, located in southwestern China, encompasses 183 counties with diverse climatic conditions. It is divided into three distinct climate zones based on variations in temperature, precipitation, and sunlight (32): (a) Central Subtropical Humid Climate Zone (Zone 1): Covering 128 counties, this zone is characterized by warm, humid conditions year-round. Average annual temperatures range from 16°C to 18°C, with mild winters and hot summers. Rainfall is abundant, averaging 1,000–1,200 mm annually. Over half of the precipitation occurs during the summer months. (b) Subtropical Semi-Humid Climate Zone (Zone 2): This zone includes 23 counties and features relatively high temperatures throughout the year, averaging 12°C to 20°C. The region experiences a pronounced dry season lasting 7 months, with annual precipitation of 900–1,200 mm, 90% of which falls between May and October. (c) Plateau Cold Climate Zone (Zone 3): Comprising 32 counties, this zone is marked by significant elevation changes and a cold temperate climate. Average annual temperatures range from 4°C to 12°C, with cool summers and cold winters. Annual precipitation is lower, ranging from 500 to 900 mm, but the region benefits from ample sunshine (Figure 1).

### 2.2 Data collection

In China, BD is classified as a Category B notifiable infectious disease, requiring reporting to the local Center for Disease Control and Prevention within 24 h of diagnosis. The BD case data in this study were obtained from the National Notifiable Diseases Reporting System. Case definitions adhered to the standardized criteria established by the National Health and Family Planning Commission of the People’s Republic of China.^1^ Both clinically diagnosed and laboratory-confirmed cases were included in the analysis. Meteorological and ecological data were sourced from the National Earth System Science Data Center, National Science and Technology Infrastructure of China^2^ and the National Tibetan Plateau/Third Pole Environment Data Center.^3^ Meteorological factors: precipitation, average temperature, minimum temperature, maximum temperature (33), PM10 (34), and potential evaporation. Ecological factors: vegetation cover (250 m) (35) and NDVI. These factors, along with case numbers, were matched with county-level administrative divisions to construct a county-level BD database for Sichuan Province spanning January 2005 to December 2023. All case data in this study were anonymized and did not require institutional review board assessment.

### 2.3 Data analysis

In this study, we employed the XGBoost machine learning model. First, we conducted a fitting analysis on the province-wide data. Subsequently, we categorized the data according to different climate zones and conducted separate model fitting analyses for each climate zone. The specific steps were as follows: To ensure reliable evaluation, we used stratified sampling to split the dataset into a training set (70%) and a test set (30%). Hyperparameter tuning and model evaluation were carried out using 10-fold cross-validation and Bayesian optimization (detailed hyperparameter settings are provided in Supplementary Table 1). Model performance was evaluated using Root Mean Square Error (RMSE), Mean Absolute Error (MAE), and R-squared (R^2^). Additionally, SHAP (SHapley Additive exPlanations) analysis was applied to interpret the model and quantify the contribution of each variable to the predictive outcomes. To assess the lag effects of the variables, we conducted lag effect analyses on the two most important variables, with lag periods set to 1 to 3 months. MAE was used as the evaluation metric to measure the lagged impact of these variables on model performance. All procedures were implemented in Python version 3.12.4,^4^ with the spatial distribution map created using QGIS version 3.40.0.^5^

## 3 Results

### 3.1 Descriptive analysis

**Figure 2:**
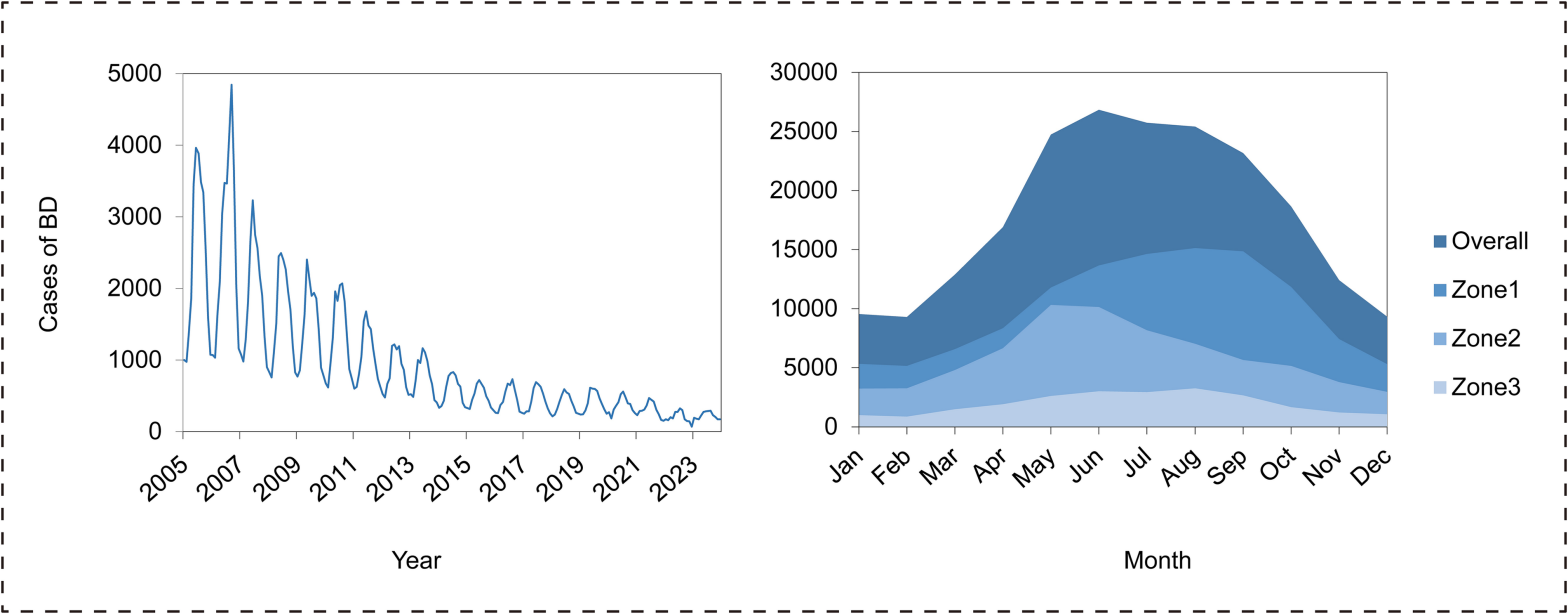
Time series distribution of bacillary dysentery (BD) cases and monthly case distribution by region: Overall (province-wide), Zone 1 (Central Subtropical Humid Climate Zone), Zone 2 (Subtropical Semi-Humid Climate Zone), and Zone 3 (Plateau Cold Climate Zone).

Between 2005 and 2023, Sichuan Province exhibited considerable variability in BD case counts, ultimately demonstrating a general decrease. The yearly incidence rate decreased from 34.72 to 3.25 per 100,000 individuals, exhibiting significant seasonal variation: 67.25% of incidences transpired between May and October, with a peak in June. The peak incidence periods varied across different climate zones: the Central Subtropical Humid Climate Zone peaked from August to September, the Subtropical Semi-Humid Climate Zone peaked from May to June, and the Plateau Cold Climate Zone peaked from July to August (Figure 2). Cases were documented in all 183 counties, albeit the distribution exhibited geographical variation. The Central Subtropical Humid Climate Zone included 55.86% of cases (mean: 938 cases per county), succeeded by the Subtropical Semi-Humid Climate Zone (33.11%; 3,094 cases per county) and the Plateau Cold Climate Zone (11.03%; 741 cases per county) (Supplementary Figure 1). We performed statistical calculations on meteorological and ecological data from 183 counties. Key metrics, including the mean, median, and standard deviation, were obtained for eight factors: precipitation, average temperature, minimum temperature, maximum temperature, PM10, potential evaporation, NDVI, and vegetation cover (250 m). Detailed results are provided in Supplementary Table 2.

### 3.2 Performance of machine learning models

Using monthly data from 2005 to 2023, we conducted XGBoost model analyses for the entire province as well as for each individual climate zone. The performance metrics of the models on the test set were presented in Table 1. The MAE values for the overall (province-wide), Zone 1, Zone 2, and Zone 3 were 4.40, 3.21, 10.87, and 2.77, respectively, while the RMSE values were 9.96, 6.05, 20.21, and 5.30, reflecting the average and overall error levels of the models. The R^2^ values, representing the models’ explanatory power, were 0.76, 0.89, 0.77, and 0.81, respectively. In summary, the models demonstrated strong predictive performance.

**Table 1:** Performance metrics of the XGBoost model across climate zones.

Metrics	Overall	Zone 1	Zone 2	Zone 3
MAE	4.40	3.21	10.87	2.77
RMSE	9.96	6.05	20.21	5.30
R2	0.76	0.89	0.77	0.81

### 3.3 Feature analysis

**Figure 3:**
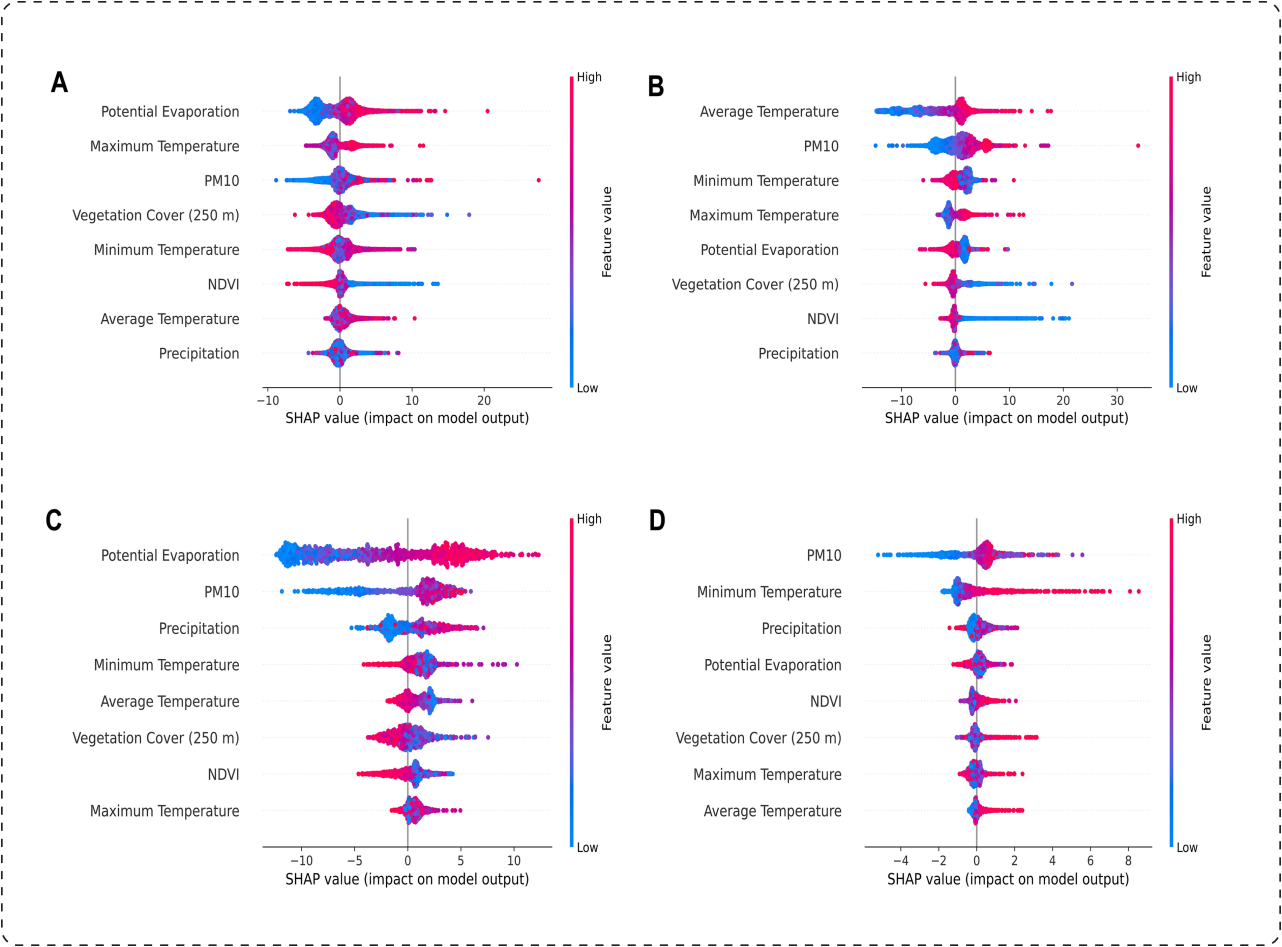
Distribution of SHAP values for environmental factors across regions: **(A)** Overall, **(B)** Zone 1, **(C)** Zone 2, and **(D)** Zone 3.

**Figure 4:**
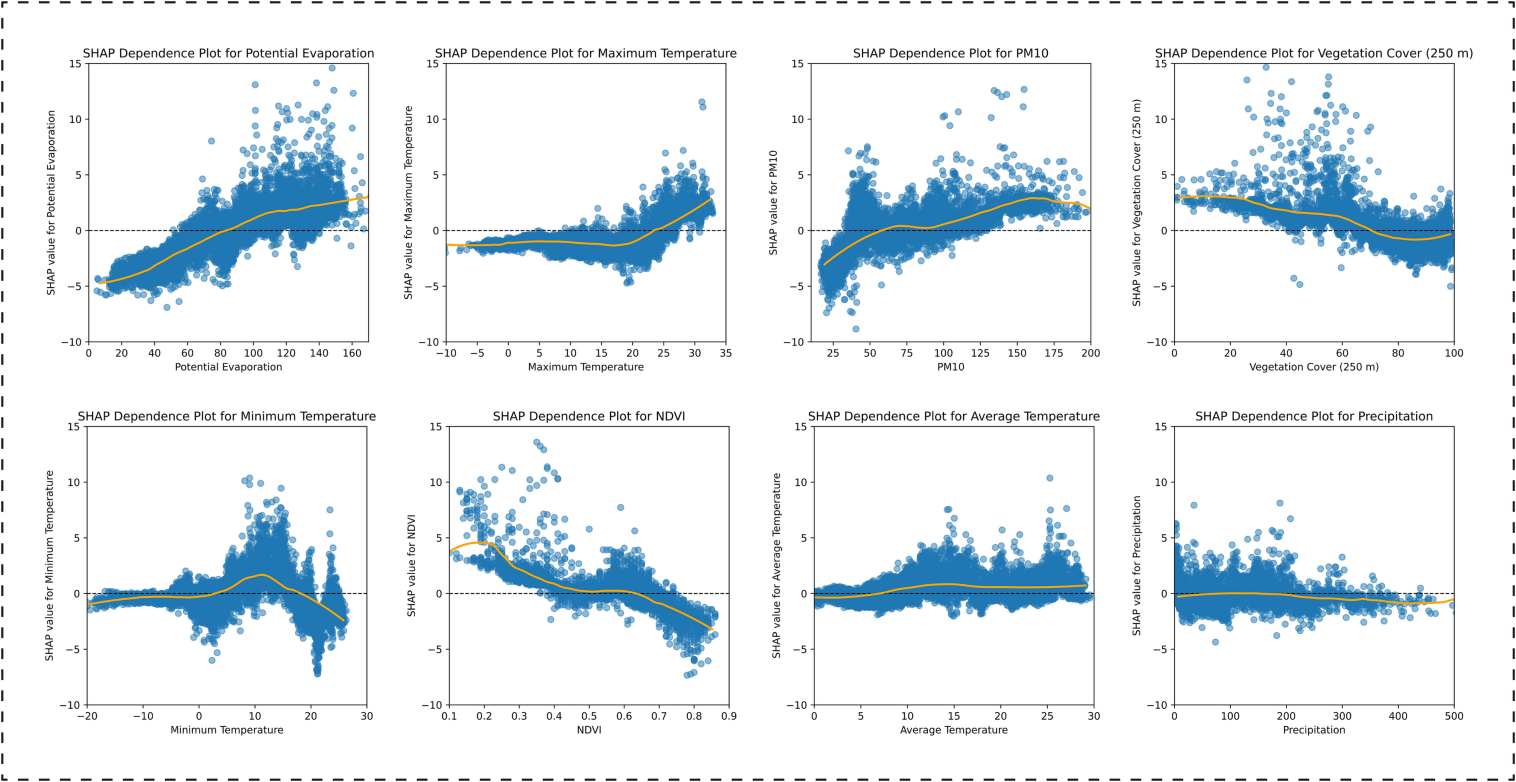
SHAP dependence plots for environmental features in the XGBoost model: Province-wide analysis.

Through SHAP analysis, this study systematically revealed the importance ranking of the influencing factors included in the model (see Supplementary Table 3 for details) and effectively distinguished the positive and negative correlations between these factors and BD incidence (Figure 3). Province-wide, the factor importance ranking was as follows: potential evaporation, maximum temperature, PM10, vegetation cover (250 m), minimum temperature, NDVI, average temperature, and precipitation. Among these, potential evaporation, maximum temperature, and PM10 showed positive associations with BD incidence, while vegetation cover (250 m) and NDVI exhibited negative associations (Figure 4).

**Figure 5:**
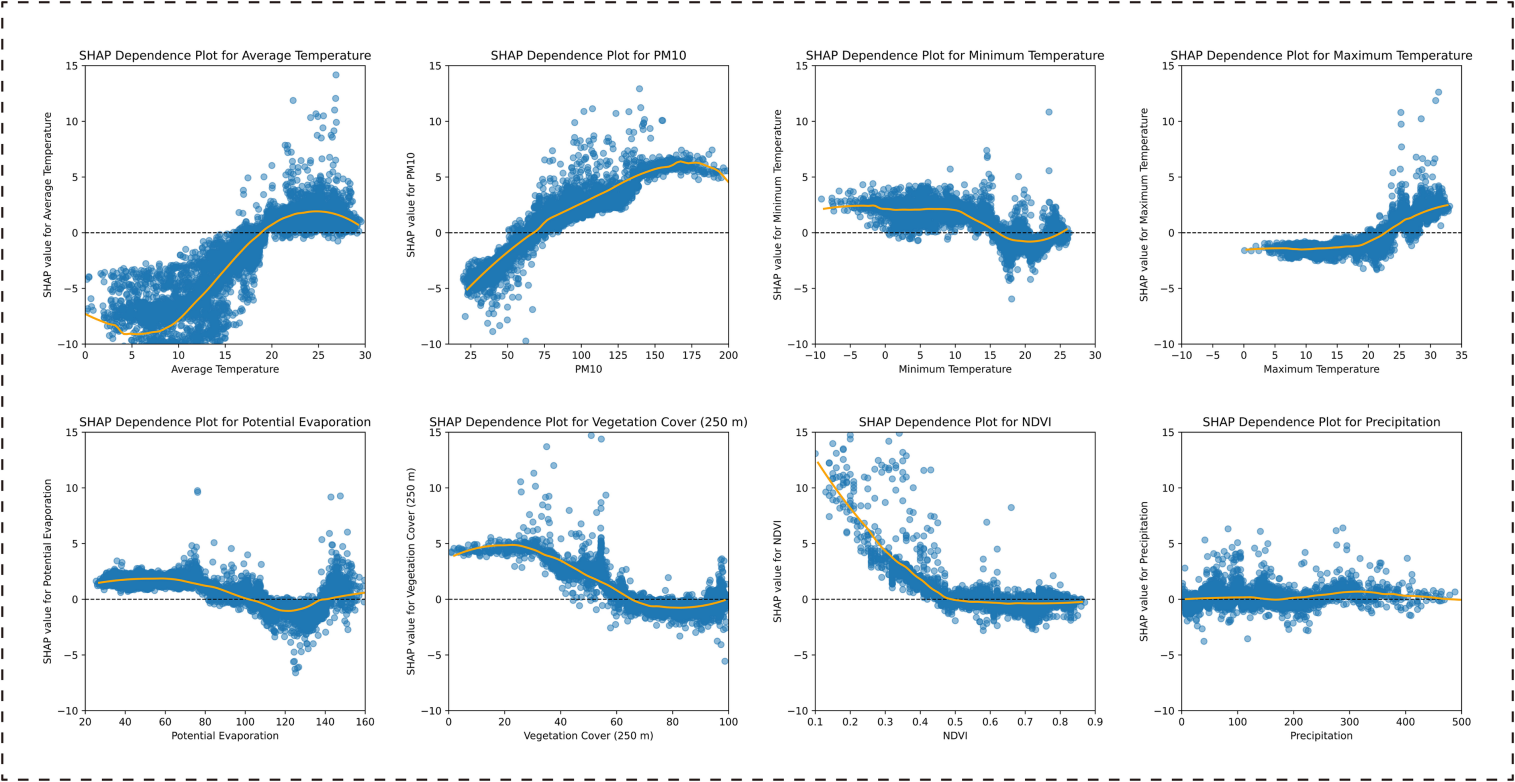
SHAP dependence plots for environmental features in the XGBoost model: Central Subtropical Humid Climate Zone analysis.

**Figure 6:**
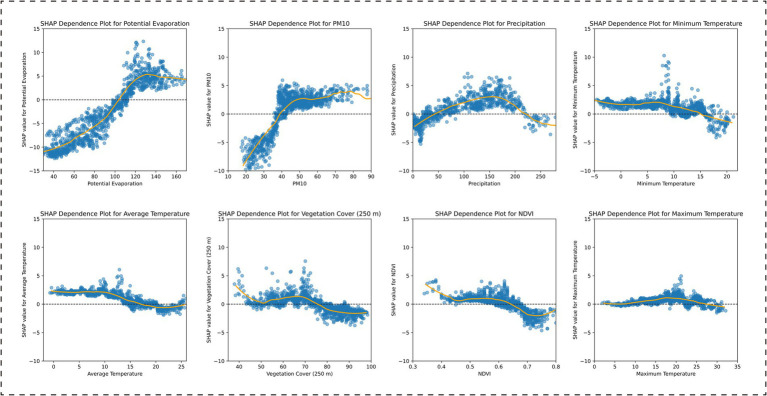
SHAP dependence plots for environmental features in the XGBoost model: Subtropical Semi-Humid Climate Zone analysis.

**Figure 7:**
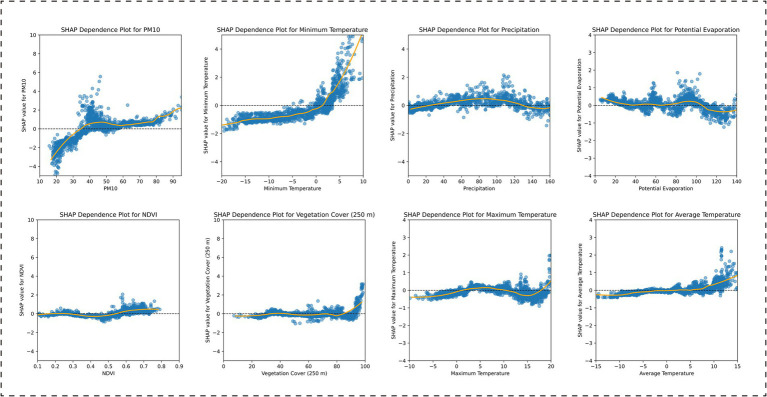
SHAP dependence plots for environmental features in the XGBoost model: Plateau Cold Climate Zone analysis.

Based on the SHAP analysis of the climate zone-specific models, the results indicated the following: In the Central Subtropical Humid Climate Zone, the factor importance ranking was: average temperature, PM10, minimum temperature, maximum temperature, potential evaporation, vegetation cover (250 m), NDVI, and precipitation. Among these, average temperature, PM10, and maximum temperature were positively correlated with BD incidence, while vegetation cover (250 m) and NDVI were negatively correlated (Figure 5). In the Subtropical Semi-Humid Climate Zone, the factor importance ranking was: potential evaporation, PM10, precipitation, minimum temperature, average temperature, vegetation cover (250 m), NDVI, and maximum temperature. PM10 and potential evaporation showed positive associations, while vegetation cover (250 m) and NDVI remained negatively correlated (Figure 6). In the Plateau Cold Climate Zone, the factor importance ranking was: PM10, minimum temperature, precipitation, potential evaporation, NDVI, vegetation cover (250 m), maximum temperature, and average temperature. Minimum temperature, average temperature, and PM10 were positively correlated with BD incidence, but no significant negative associations were detected (Figure 7). The lagged effect analysis (1–3 months) for the top two most important variables revealed minimal impacts of different lag periods on the model’s MAE values (see Supplementary Table 4 for details). Furthermore, to further quantify the relationships between influencing factors and BD incidence, this study analyzed the threshold effects of key environmental parameters using SHAP dependence curves. The results showed that the interaction patterns of these factors across climate zones were highly consistent with the findings above. Detailed threshold values will be thoroughly discussed in the subsequent analysis section.

## 4 Discussion

Based on long-term, fine-scale research data, this study systematically revealed the spatiotemporal heterogeneity of BD incidence and its environmental driving mechanisms across different climate zones in Sichuan Province. Analysis using the XGBoost machine learning model demonstrated that environmental factors significantly influenced BD transmission in a climate zone-specific manner, highlighting the importance of fine-resolution climate zoning in assessing BD incidence risk.

To elucidate the contribution of each environmental factor, this study employed the game theory-based SHAP analysis method. The core principle of this method involves calculating Shapley values to quantify the contribution of each feature to the prediction outcome, thereby enhancing model transparency and interpretability. This approach assists researchers and policymakers in gaining a deeper understanding of how various factors influence prediction results, providing valuable insights for BD monitoring and prevention.

The association between ambient temperature and BD incidence exhibited significant spatial heterogeneity. In the Central Subtropical Humid Climate Zone, BD incidence risk significantly increased when the average temperature exceeded a threshold of 18°C, which aligns with the growth temperature of *Shigella* (6–8°C to 45–47°C, optimal around 37°C) (36), suggesting that warm environments facilitate bacterial proliferation and transmission both within and outside hosts. In the Plateau Cold Climate Zone, risk increased when the minimum temperature exceeded 2°C, a phenomenon that may not directly stem from pathogen biological characteristics but rather be associated with increased outdoor activities among local residents at this temperature threshold (37). Rising temperatures prompted local residents to engage in more outdoor activities, thereby increasing the risk of pathogen exposure. Conversely, in the Subtropical Semi-Humid Climate Zone, the predictive importance of temperature factors significantly decreased due to consistently high annual temperatures, suggesting that temperature is no longer a primary limiting factor for BD transmission in this region, and disease spread may be more regulated by other environmental factors, such as water availability or precipitation patterns.

The impact of precipitation on BD incidence also exhibited significant climate zone dependence. In the Central Subtropical Humid Climate Zone, well-developed sanitation infrastructure effectively blocked precipitation-related waterborne transmission routes, resulting in no clear correlation between precipitation and BD incidence. This finding highlights that robust water, sanitation, and hygiene (WASH) infrastructure can effectively mitigate the influence of climatic factors (e.g., heavy rainfall) on waterborne diseases, decoupling environmental triggers from disease outcomes. In contrast, in the Subtropical Semi-Humid Climate Zone, a sharp increase in precipitation after the dry season (monthly precipitation > 50 mm in May) coincided significantly with the peak BD incidence period. This pattern is consistent with the outbreaks of diarrheal diseases triggered by heavy rainfall after droughts observed in other global regions [e.g., Ecuador (38), Eswatini (39), the United Kingdom (40), Japan (41), and Vietnam (7)]. This may be related to the burst of microbial activity caused by rewetting dry soil (42), which, during heavy rainfall, facilitates the flushing of pathogens into water bodies, subsequently triggering disease outbreaks. This phenomenon suggests that in regions prone to alternating dry and heavy rainfall periods, public health strategies should not only focus on immediate flood response but also manage the environmental consequences of drought. In the Plateau Cold Climate Zone, BD incidence risk significantly increased when monthly precipitation exceeded 30 mm. This threshold may reflect a critical point of the region’s sanitation infrastructure capacity. Heavy rainfall can overload wastewater treatment plants, leading to the overflow of untreated or partially treated sewage into the environment, or overwhelm septic tank systems, increasing the risk of fecal contaminant entry into water sources. This suggests that even moderate precipitation can pose a public health threat in regions with relatively weak infrastructure.

Potential evapotranspiration exhibited the highest predictive importance in the Subtropical Semi-Humid Climate Zone. As a comprehensive indicator reflecting meteorological factors such as temperature, humidity, wind speed, and solar radiation, an increase in potential evaporation was associated with the synergistic effects of high temperature, low humidity, and strong solar radiation (43). High potential evaporation values indicate increased atmospheric demand for moisture, which, when precipitation is insufficient, exacerbates regional water stress. Under these circumstances, residents may be forced to rely on suboptimal or unsafe water sources, thereby increasing their risk of exposure to pathogenic microorganisms. As a comprehensive meteorological indicator, potential evaporation can serve as a valuable early warning indicator for hydrological stress and impending water scarcity, supporting early warning systems for waterborne disease risks.

Increased PM10 concentration were positively correlated with BD incidence. Based on existing research, this study proposes that PM10 may influence BD incidence through the following mechanisms: First, particulate matter can serve as a carrier for pathogenic microorganisms (44), facilitating their direct transmission through aerosol deposition or water contamination. Second, PM10 can alter the local microenvironment, affecting pathogen survival and transmission efficiency. Chemical pollutants within particulate matter may inhibit microbial growth at high concentrations but could provide a suitable microenvironment and nutrients at moderate concentrations (45). Furthermore, long-term exposure to PM10 may impair the barrier function of the intestinal mucosa, thereby increasing host susceptibility to pathogens. PM10 exposure can lead to alterations in the gut microbiota, reducing the abundance of beneficial microorganisms and promoting the overgrowth of pro-inflammatory species, which in turn contributes to intestinal barrier dysfunction, oxidative stress, and inflammatory responses, all associated with the development and progression of gastrointestinal inflammatory diseases (46, 47).

Ecological factors, including vegetation cover (250 m) and NDVI, were negatively correlated with BD incidence in both the Central Subtropical and Subtropical Climate Zones. These results have been corroborated in previous studies (48, 49). This study hypothesizes that the mechanisms by which green spaces reduce BD incidence may include: Vegetation, particularly riparian vegetation, reduces runoff of sediments, fertilizers, and pesticides from agricultural fields through physical buffering, and helps capture and cycle nutrients, preventing their excessive entry into water bodies that could lead to eutrophication (50). Furthermore, vegetation can lower ambient temperatures by providing shade and enhancing evapotranspiration, thereby mitigating the risk of BD transmission associated with rising temperatures (51, 52).

Furthermore, this study also found that BD case numbers in Sichuan Province exhibited significant seasonal variations, with differing incidence peaks across climate zones, suggesting region-specific seasonal driving mechanisms. In the Central Subtropical Humid Climate Zone, the peak BD incidence occurred from August to September. This correlated with the average temperature in this region exceeding the 18°C threshold. Despite the well-developed sanitation infrastructure in this region, which effectively blocked direct waterborne transmission routes related to precipitation, high summer temperatures may promote pathogen transmission by accelerating bacterial growth and increasing the frequency of outdoor activities and water body contact. In the Subtropical Semi-Humid Climate Zone, the peak BD incidence occurred from May to June. This peak period significantly coincided with a sharp increase in precipitation after the dry season (monthly precipitation >50 mm in May), indicating that this ‘dry-wet transition’ effect was a key driving factor for seasonal BD outbreaks in this region (42). Additionally, this region may experience water stress, compelling residents to rely on suboptimal water sources during the dry season, while runoff pollution from initial rainfall in the wet season exacerbates the incidence risk. In the Plateau Cold Climate Zone, the peak BD incidence occurred from July to August. This correlated with the minimum temperature in this region exceeding the 2°C threshold, where rising temperatures may encourage local residents to increase outdoor activities. Furthermore, increased monthly precipitation may also contribute to regulating seasonal BD incidence in this region. These climate zone-specific seasonal patterns further emphasize that BD epidemiology is not determined by a single factor, but rather results from complex interactions among environmental conditions, infrastructure, and human behavior. Understanding these refined seasonal driving mechanisms is crucial for developing more targeted and timely public health interventions.

Despite utilizing the XGBoost model to elucidate the influence of meteorological and ecological factors on BD incidence, this study has several limitations, specifically: First, no statistically significant lagged effects were identified in this study; however, this does not imply the absence of lagged impacts of environmental factors on BD incidence. The inconspicuous lagged effects may be attributed to inherent limitations of the current dataset and modeling methodology. Second, the model relies on reported data, which may be subject to underreporting issues. Third, BD transmission is also influenced by socioeconomic factors, sanitation infrastructure, and public health awareness (53, 54), which were not fully considered in this study. Finally, while SHAP analysis effectively identified key predictive factors, the method itself only captures associations between features and predicted outputs, without establishing causal relationships. Therefore, the results of this study should be interpreted with caution.

In conclusion, through an in-depth analysis of the spatiotemporal heterogeneity of BD and its environmental driving mechanisms in Sichuan Province, this study revealed the influence of environmental factors on BD transmission. These findings provide a scientific basis for developing climate zone-specific BD monitoring, prevention, and intervention strategies. In the future, a more comprehensive BD surveillance and reporting system should be established, integrating socioeconomic factors to more comprehensively assess the potential influencing factors of BD incidence, thereby achieving more precise and effective disease control and prevention.

## Data Availability

The raw data supporting the conclusions of this article will be made available by the authors, without undue reservation.

## Footnotes

^1^ http://www.nhc.gov.cn/zwgkzt/s9491/200802/39040.shtml

^2^ http://www.geodata.cn

^3^ http://data.tpdc.ac.cn

^4^ https://www.python.org/downloads/release/python-3124/

^5^ https://www.qgis.org/

## Supplementary material

SUPPLEMENTARY FIGURE 1 Temporal distribution of BD cases across counties in Sichuan Province.
