# Supplementary material for: Interpretable machine learning analysis of environmental characteristics on bacillary dysentery in Sichuan Province
Source: Front Public Health. 2025 Jul 16;13:1598247. doi: 10.3389/fpubh.2025.1598247 (PMC12307499; doi:10.3389/fpubh.2025.1598247)
Supplement: SUPPLEMENTARY FIGURE 1 — Temporal distribution of BD cases across counties in Sichuan Province. [file Table_1.docx]

Supplementary Table 1. Optimized hyperparameters of the XGBoost model by climate zone

| **Parameter** | **Description** | **Overall** | **Zone 1** | **Zone 2** | **Zone 3** |
| --- | --- | --- | --- | --- | --- |
| colsample_bytree | Fraction of features used for building trees | 1.0 | 1.0 | 1.0 | 1.0 |
| gamma | Minimum loss reduction for a split | 1.96 | 5.0 | 4.86 | 0.0 |
| learning_rate | Step size shrinkage to prevent overfitting | 0.01 | 0.01 | 0.01 | 0.02 |
| max_depth | Maximum depth of a tree | 10 | 7 | 9 | 10 |
| min_child_weight | Minimum sum of instance weights in a child node | 10 | 2 | 10 | 10 |
| n_estimators | Number of trees in the model | 1033 | 1877 | 195 | 100 |
| subsample | Fraction of samples used for fitting trees | 0.6 | 0.6 | 0.6 | 0.6 |

Supplementary Table 2. Descriptive statistics of monthly meteorological and ecological factors (2005–2023)

| **Feature** | | **Mean** | **Median** | **Mode** | **Standard Deviation** | **Variance** | **Q1** | **Q3** |
| --- | --- | --- | --- | --- | --- | --- | --- | --- |
| Overall | Precipitation（mm） | 86.44 | 60.43 | 5.30 | 83.83 | 7027.33 | 17.36 | 134.99 |
|  | Average Temperature（℃） | 13.59 | 13.84 | 7.31 | 9.01 | 81.12 | 7.47 | 21.20 |
|  | Minimum Temperature（℃） | 9.26 | 9.71 | 15.27 | 9.63 | 92.67 | 3.15 | 16.98 |
|  | Maximum Temperature（℃） | 17.88 | 18.10 | 11.32 | 8.45 | 71.40 | 11.51 | 25.16 |
|  | PM10（µg/m³） | 70.87 | 63.90 | 67.62 | 34.84 | 1214.14 | 44.42 | 90.02 |
|  | Potential Evaporation（mm） | 84.39 | 84.33 | 44.60 | 38.51 | 1482.70 | 47.53 | 120.28 |
|  | NDVI | 0.56 | 0.58 | 0.61 | 0.13 | 0.02 | 0.48 | 0.66 |
|  | Vegetation Cover (250 m)（%） | 69.65 | 71.54 | 72.82 | 19.05 | 362.99 | 56.77 | 85.44 |
| Zone 1 | Precipitation（mm） | 95.43 | 67.08 | 14.08 | 89.59 | 8027.03 | 21.93 | 146.50 |
|  | Average Temperature（℃） | 16.61 | 17.40 | 24.71 | 7.30 | 53.32 | 9.96 | 22.92 |
|  | Minimum Temperature（℃） | 12.78 | 13.54 | 15.27 | 7.48 | 55.96 | 6.04 | 19.19 |
|  | Maximum Temperature（℃） | 20.33 | 20.92 | 26.78 | 7.21 | 51.94 | 14.12 | 26.67 |
|  | PM10（µg/m³） | 81.19 | 76.28 | 67.62 | 35.57 | 1265.49 | 54.26 | 102.49 |
|  | Potential Evaporation（mm） | 88.04 | 87.90 | 44.30 | 38.21 | 1459.89 | 48.59 | 125.51 |
|  | NDVI | 0.57 | 0.58 | 0.61 | 0.12 | 0.02 | 0.49 | 0.66 |
|  | Vegetation Cover (250 m)（%） | 71.12 | 73.37 | 95.85 | 19.18 | 367.87 | 58.90 | 87.06 |
| Zone 2 | Precipitation（mm） | 74.35 | 44.06 | 4.28 | 74.25 | 5512.82 | 9.85 | 134.18 |
|  | Average Temperature（℃） | 13.11 | 14.00 | 16.75 | 6.10 | 37.18 | 8.33 | 17.73 |
|  | Minimum Temperature（℃） | 8.42 | 9.31 | 8.96 | 6.03 | 36.32 | 3.86 | 12.88 |
|  | Maximum Temperature（℃） | 18.05 | 18.83 | 20.22 | 6.27 | 39.33 | 13.31 | 22.91 |
|  | PM10（µg/m³） | 45.57 | 44.81 | 49.11 | 14.04 | 197.08 | 36.22 | 53.59 |
|  | Potential Evaporation（mm） | 90.58 | 89.80 | 50.01 | 35.44 | 1255.72 | 57.49 | 120.64 |
|  | NDVI | 0.60 | 0.61 | 0.67 | 0.09 | 0.01 | 0.53 | 0.67 |
|  | Vegetation Cover (250 m)（%） | 72.29 | 72.65 | 85.76 | 13.96 | 194.86 | 62.21 | 83.56 |
| Zone 3 | Precipitation（mm） | 59.15 | 43.99 | 4.67 | 54.26 | 2943.94 | 8.26 | 103.78 |
|  | Average Temperature（℃） | 1.85 | 1.99 | 8.87 | 7.04 | 49.51 | -4.23 | 8.27 |
|  | Minimum Temperature（℃） | -4.19 | -3.98 | 3.47 | 7.02 | 49.26 | -10.18 | 1.96 |
|  | Maximum Temperature（℃） | 7.98 | 8.19 | 3.04 | 7.09 | 50.26 | 1.90 | 14.37 |
|  | PM10（µg/m³） | 47.78 | 46.80 | 43.08 | 17.90 | 320.56 | 33.71 | 61.11 |
|  | Potential Evaporation（mm） | 65.37 | 59.79 | 25.77 | 35.98 | 1294.77 | 30.69 | 96.35 |
|  | NDVI | 0.50 | 0.51 | 0.59 | 0.13 | 0.02 | 0.41 | 0.60 |
|  | Vegetation Cover (250 m)（%） | 61.91 | 60.55 | 72.20 | 19.77 | 390.74 | 46.46 | 78.44 |

Supplementary Table 3. Environmental feature importance scores derived from SHAP analysis in XGBoost models by climate zone

| **Overall** | |  | **Zone 1** | |  | **Zone 2** | |  | **Zone 3** | |
| --- | --- | --- | --- | --- | --- | --- | --- | --- | --- | --- |
| Feature | importance |  | Feature | importance |  | Feature | importance |  | Feature | importance |
| Potential Evaporation | 0.238 |  | Average Temperature | 0.320 |  | Potential Evaporation | 0.354 |  | PM10 | 0.285 |
| Maximum Temperature | 0.138 |  | PM10 | 0.190 |  | PM10 | 0.192 |  | Minimum Temperature | 0.281 |
| PM10 | 0.133 |  | Minimum Temperature | 0.111 |  | Precipitation | 0.111 |  | Precipitation | 0.089 |
| Vegetation Cover (250 m) | 0.132 |  | Maximum Temperature | 0.104 |  | Minimum Temperature | 0.086 |  | Potential Evaporation | 0.078 |
| Minimum Temperature | 0.124 |  | Potential Evaporation | 0.097 |  | Average Temperature | 0.073 |  | NDVI | 0.074 |
| NDVI | 0.085 |  | Vegetation Cover (250 m) | 0.086 |  | Vegetation Cover (250 m) | 0.071 |  | Vegetation Cover (250 m) | 0.070 |
| Average Temperature | 0.080 |  | NDVI | 0.059 |  | NDVI | 0.068 |  | Maximum Temperature | 0.067 |
| Precipitation | 0.071 |  | Precipitation | 0.033 |  | Maximum Temperature | 0.044 |  | Average Temperature | 0.056 |

Supplementary Table 4. MAE in different zones with different time delay

| Feature | | MAE | | | |
| --- | --- | --- | --- | --- | --- |
|  |  | No delay | One-month delay | Two-month delay | Three-month delay |
| Overall | Potential Evaporation | 4.40 | 4.35 | 4.38 | 4.37 |
|  | Maximum Temperature | 4.40 | 4.32 | 4.34 | 4.32 |
| Zone 1 | Average Temperature | 3.21 | 3.21 | 3.17 | 3.18 |
|  | PM10 | 3.21 | 3.21 | 3.20 | 3.22 |
| Zone 2 | Potential Evaporation | 10.87 | 10.73 | 10.78 | 10.88 |
|  | PM10 | 10.87 | 11.23 | 10.67 | 10.65 |
| Zone 3 | PM10 | 2.77 | 2.91 | 2.83 | 2.81 |
|  | Minimum Temperature | 2.77 | 2.82 | 2.85 | 2.73 |
